# Potential therapeutic targets of triple-negative breast cancer based on its intrinsic subtype

**DOI:** 10.18632/oncotarget.20274

**Published:** 2017-08-16

**Authors:** Fangyuan Shao, Heng Sun, Chu-Xia Deng

**Affiliations:** ^1^ Faculty of Health Sciences, University of Macau, Macau SAR, China

**Keywords:** cancer therapy, TNBC subtype, CSCs, drug resistance, cancer targets

## Abstract

Triple-negative breast cancer (TNBC) is an aggressive subgroup of human breast cancer, which is characterized as estrogen receptor (ER) negative, progesterone receptor (PR) negative, and human epidermal growth factor receptor 2 (HER2) negative. TNBC is the most difficult breast cancer subgroup to treat, due to its unresponsiveness to current clinical targeted therapies, high rate of recurrence, and poor prognosis. Thus, there is an urgent medical need to identify therapeutic targets and develop more effective stratified medicine for the treatment of TNBC. Here we review the potential therapeutic targets for TNBC based on its intrinsic subtype. We also review the aberrant activated signals found in different subgroups of TNBC, including androgen receptor (AR) and PI3K/AKT/mTOR, Notch, Wnt/β-catenin, Hedge-hog, and TGF-β signaling pathways, which play essential roles in multiple development stages of TNBC. The careful analysis of these signaling pathways and therapeutic targets would have significant impact on the drug development and clinical trials, leading to effective therapies for this deadly disease.

## INTRODUCTION

Triple-negative breast cancer (TNBC) accounts for approximately 15%–20% of newly diagnosed breast cancer worldwide and occurs more prevalently in Hispanics, American and African women [1–3]. In fact, TNBC is a class of highly heterogeneous tumors incorporating various molecular and clinic pathologic features and clinical outcomes. Approximately 80% of TNBC overlaps with the basal-like breast cancer phenotype that is classified by gene expression profiling, although TNBC and basal-like breast cancer are never synonymous [4]. In general, TNBC is more destructive, with higher rates of relapse compared to other types of breast cancer, and shows frequently metastasis to the visceral and central nervous system [5]. In the absence of obvious targets, which could benefit pharmaceutical development, TNBC cases are mainly treated by tumor excision, radiation therapy and chemotherapy using cytotoxic agents. A lot of well-established target therapies that have been approved for the treatment of other cancers are found to be beneficial in the case of TNBC, including agents that target Poly [ADP ribose] polymerase 1 (PARP1) [6], androgen receptor (AR) [7], vascular endothelial growth factor receptor (VEGFR) and epidermal growth factor receptor (EGFR) under certain circumstances [8].

Although multiple targeted agents and monoclonal antibodies are under investigation for TNBCs, the majority of them have failed to achieve a satisfying therapeutic outcome. The lack of biomarkers to stratify TNBC and identify sensitive patients who are most likely to respond to different targeted therapy remains a major reason for these disappointing results. Here, we have reviewed different studies which classify TNBC into subtypes, and investigated representative therapeutic strategies for these subtypes.

## FURTHER SUBTYPING OF TNBC

**Figure 1:**
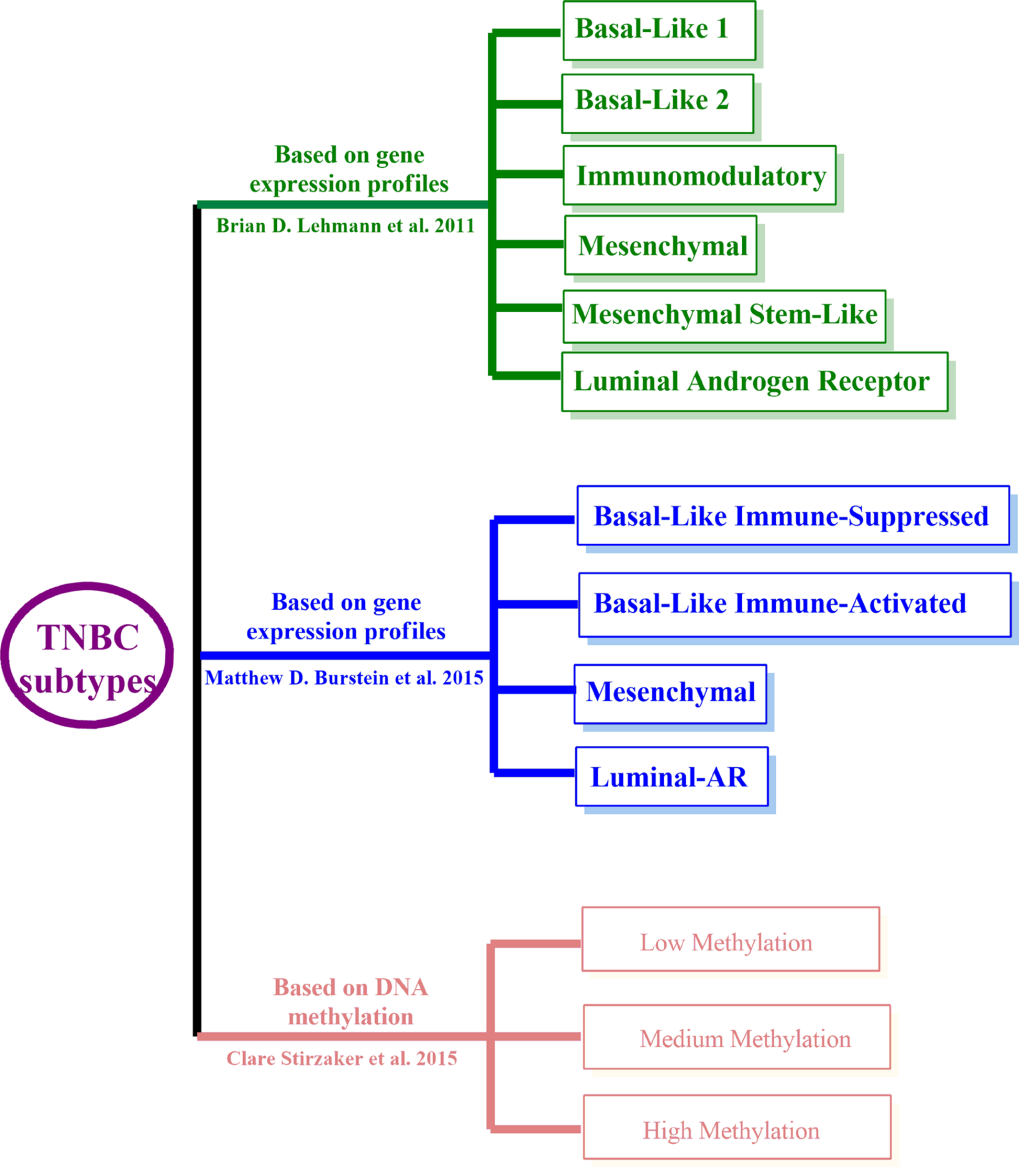
TNBC classifications Brian's (Green), Matthew's (Blue), and Clare's methylation subtyping (Red).

Vast advances have been made to classify TNBC into more molecular subtypes, which have enormous potential for personalized medicine and guidance for clinical trial. The pioneer studies were conducted by Brian et al. (Figure 1), who reported six TNBC subtypes based on gene expression profiling (GEP), i.e., basal-like 1 (BL1), basal-like 2 (BL2), an immunomodulatory (IM), a mesenchymal (M), a mesenchymal stem-like (MSL), and a luminal androgen receptor (LAR) subtype [9]. More importantly, they identified representative TNBC cell lines of these subtypes to show that analysis of distinct gene expression profiles can inform therapy selection. BL-TNBC is characterized by DNA-repair deficiency, and the relevant cell models responded to cisplatin treatment. The M and MSL subtypes have higher expression of genes involved in epithelial to mesenchymal transition (EMT) and activation of receptor tyrosine kinase pathways, and representative cell lines preferentially responded to PI3K/mTOR inhibitors and ABL/SRC inhibitors. The LAR subtype is characterized by androgen receptor signaling and LAR cell lines were uniquely sensitive to AR antagonists.

More recently, Matthew et al. revisited the grouping of TNBC by analyzing the RNA and DNA profile of 198 TNBC tumors, and classed these TNBCs into four, rather than six subtypes (Figure 1), i.e., Luminal-AR (LAR), Mesenchymal (MES), Basal-Like Immune-Suppressed (BLIS), Basal-Like Immune-Activated (BLIA) [10]. They also identified putative subtype-specific targets: androgen receptor and the cell surface mucin MUC1 for the LAR subtype; PDGF receptor A and c-Kit for the MES subtype; VTCN1, an immune suppressing molecule for the BLIS subtype; and STAT signal transduction molecules and cytokines for the BLIA subtype. These studies imply a promising future for personalized therapy in TNBC based on molecular subtype, and further studies for subtype-specific therapy are rationally warranted.

The development of a tumor is a multistep process resulting from the accumulation of genetic alterations, which involve not only changes in the DNA sequence, but also epigenetic alterations such as DNA methylation and histone modification. Recently, emerging epigenetic analyses, especially for the DNA methylation, have contributed to the further stratification of TNBC. Clare et al. tested whether DNA methylation signatures can classify TNBC, and whether they could be used to predict specific clinical outcomes [11]. A total of 25 TNBC patient tumor samples were analyzed by whole-genome methylation capture sequencing assay, and 865 differentially methylated regions (DMRs) were identified. Among them, they found 36 DMRs were specific to TNBC by comparing these with 542 samples from the TCGA breast cancer cohort. Specifically, they utilized DMRs to stratify TNBC patients into three distinct methylation clusters, which were strongly associated with overall survival (Figure 1). Using a similar strategy, 17 DMRs were identified from the TCGA breast cancer cohort, which were also associated with survival in TNBC samples.

Vast efforts have also been made to stratify breast cancer utilizing the DNA methylation patterns [12]. In the global methylation profile, TNBC (the basal-like variety in their study) was the least frequently methylated, and another study also showed nine epigenetic biomarker genes were hypo-methylated in the basal-like and claudin-low breast cancers (normally TNBC) [13].

These works indicate that DNA methylation signatures could extend our ability to classify breast cancer, which will not only improve diagnosis and prognosis of breast cancer but also help to develop new therapeutic targets, which is specific to different subgroups of TNBC.

### BASAL-LIKE TNBC

BL-TNBC is the predominant molecular subgroup of TNBC. In Brian's study, two basal-like subtypes were identified. The GEP of BL1 is enriched for cell cycle check point and DNA damage response genes, and BL2 is enriched for genes involved in growth factor pathways. Particularly, TNBC cell lines from the BL1 and BL2 groups are sensitive to cisplatin treatment. Matthew identified two basal-like subtypes (BLIA and BLIS), which have down-regulation (BLIS) or up-regulation (BLIA) in immune cell-regulating pathways and cytokine pathways. Hence, immune-based strategies may be useful treatments for BLIS tumors which will be further discussed in the “immune associated TNBC” part, and BLIA tumors are sensitive to STAT inhibitors, cytokine or cytokine receptor antibodies.

### Platinum salt drug based chemotherapy

As reported by Brian, BL1 is enriched for tumors that harbor a deficiency in (homologous recombination) HR repair, which is largely caused by mutations or epigenetic changes in the BRCA1/2; and the BL2 subgroup, on the other hand, is uniquely enriched in growth factor signaling pathways (EGF, NGF and MET pathways). Thus, targeting DNA-repair deficiency by DNA damage agents appears to be a promising treatment for BL-TNBC (more likely to be effective for BL1). Indeed, good response rates to platinum-based chemotherapy have been associated with low BRCA1-mRNA expression and high BRCA1 methylation [14, 15]. Platinum-based chemotherapy appears to significantly increase the pathological complete response (pCR) rate in TNBC patients, which is relatively higher in patients with a family history of BRCA-mutation than the rest of the population [16]. Platinum salts have been increasingly tested for TNBC in combination with various other chemotherapy drugs e.g. gemcitabine [17, 18], which masquerades as cytidine and inhibits DNA synthesis. A phase III trial involving 236 patients demonstrated that cisplatin plus gemcitabine is an alternative or even the preferred first-line chemotherapy strategy for patients with metastatic triple-negative breast cancer [19].

### Inhibition of poly (ADP ribose) polymerase 1

**Figure 2:**
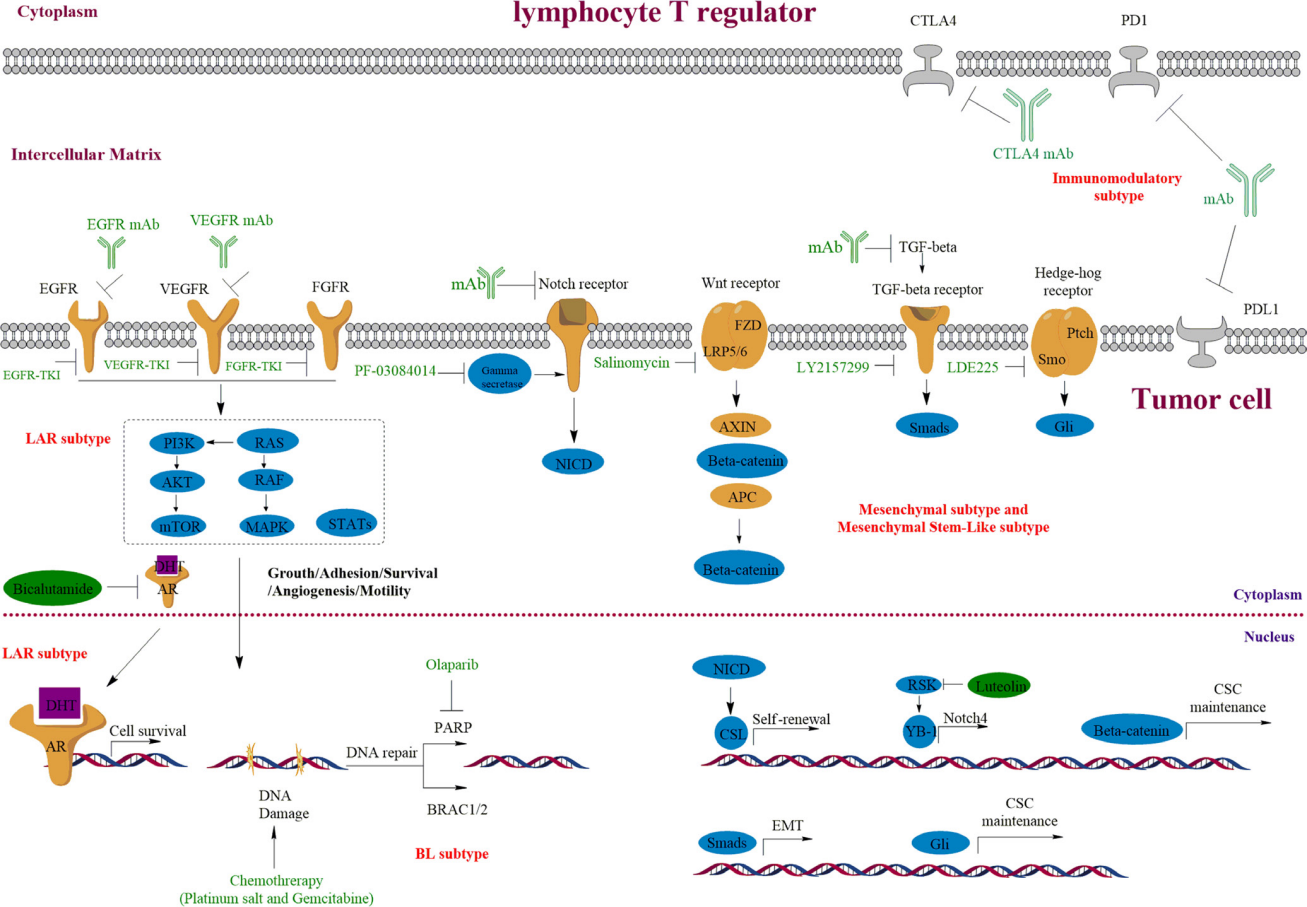
Targeting the growth factor receptors and PARP in TNBC and the important roles of Notch, Wnt/β-catenin, Hedge-hog and TGF-β signaling pathways in TNBC Overexpression or mutations of the EGFR, VEGFR, AR and FGFR are common in TNBC, which result in the deregulation of downstream signaling. Receptor specific-monoclonal antibody (mAb) and TKIs are used to block ligand-receptor interaction or kinase activity, which further turnoff their downstream signaling. The BL2 subtype of TNBC could be especially sensitive to these growth signaling inhibition. BRCA1/2 mutations or decreased expression are frequently involved in TNBC initiation and development, which also causes HR deficiency and hypersensitive to PARP inhibition (BL1 subtype). Mesenchymal-like subgroup of TNBC is enriched for genes involved in CSCs regulation and EMT, and corresponding tumors could be sensitive to mAb and inhibitors in these pathways.

Besides platinum salt-based agents, this subtype of TNBC is also believed to respond to PARP inhibitors, which cause synthetic lethal effects with HR-repair deficiency (Figure 2). PARP1 is involved in the process of responding to single-strand DNA damage, and maintains genomic integrity *via* base excision repair [20]. Double-strand DNA damages are normally repaired through HR, which requires normal functions of the tumor suppressor proteins BRCA1/2 [21]. Furthermore, it has been estimated that up to three quarters of BRCA1-associated tumors are BLBCs [22], and many TNBCs are frequently found to have defects in BRCA-related HR [23, 24]. Thus, it has provided a strong rationale for the use of PARP inhibitors for the treatment of TNBC with HR deficiency, and devoid of side-effects on the remaining normal cells. The American Society of Clinical Oncology (ASCO, 2017) state that Olaparib (PARP inhibitor) slows the growth of BRCA-related metastatic Breast Cancer. Findings suggest that such PARP inhibitors could play a key role in Breast Cancer treatment. Indeed, clinical trials showed that Olaparib significantly improved the overall survival in phase I/II clinical trials [25, 26], and Iniparib, another PARP inhibitor, is assessed in phase III clinical trials in advanced TNBC. However, these trials failed to meet the primary study end points (mainly failed in PFS and overall survival), which is thought to be due to a lack of powerful selection of BRCA1 mutated TNBC [27]. Thus, further studies are needed to better understand and target the resistance to PARP1 inhibitors. More recently, a phase III trial (the patients involved were HER2-negative metastatic breast cancer cases with a germline BRCA mutation) reported that the median progression-free survival was significantly longer in Olaparib monotherapy group than in the standard chemotherapy group, and the risk of disease progression or death was lower than for standard chemotherapy [28].

In 2006, De Soto evaluated the sensitivity of multiple cell lines (non-cancerous mouse embryonic stem cells and hamster cells; human and mouse breast tumor cells) with BRCA1 or BRCA2 deficiency to three PARP1 inhibitors (NU1025, 3-aminobenzamide, and AG14361) [29]. They showed that AG14361 has high selectivity to kill BRCA1-knockout embryonic stem cells. Whereas to human and mouse breast tumor cells tested, the PARP1 inhibitors were either ineffective or eliminate these cells irrespective of BRCA1 status. During BRCA1 mutation and carcinogenesis, the cells may go through two distinct phases according to their sensitivity to PARP1 inhibitors. In the initial stage of BRCA1 mutation, cells are generally naïve and sensitive before they acquire multiple genetic mutations and eventually develop into cancer cells, which are resistant to PARP1 inhibition. In the same year, Hochegger et al. reported that Ku-70 or Ligase IV mutation is responsible for the resistance to double-strand breaks inducing drugs in chicken DT40 cells with a heterozygous PARP1 mutation [30].

Currently, other PARP inhibitors (Veliparib and Talazoparib) are still under investigation in the cases of BRCA mutation-associated breast cancer (ClinicalTrials.gov NCT01945775 and NCT02163694), and further analyses are necessary to determine if a specific subset of TNBC patients can benefit from use of the PARP inhibitors. Olaparib was approved by Food and Drug Administration for the treatment of BRCA1-deficient ovarian cancer in 2014 [31].

## MESENCHYMAL ASSOCIATED TNBC

Mesenchymal and mesenchymal stem like TNBCs generally indicate the subgroups of TNBCs, which are enriched for genes involved in EMT and the biological regulation of CSCs (Figure 2). TNBC cell lines from the mesenchymal subgroup (e.g. MDA-MB-231) are highly resistant to multiple cytotoxic agents and possess stem cell phenotypes.

Human breast cancer cells with CD44^+^/CD24^−^/EpCAM^+^ expression markers and/or possessing ALDH1 enzyme activity are recognized as CSCs and are responsible for maintaining tumor growth. Unlike other cells within the tumor bulk, these cells are commonly insensitive to current chemotherapies [32]. Over-expression of multidrug resistance proteins [33] and anti-apoptotic proteins [34] are responsible for the resistance to some cytotoxic agents. It's widely recognized that the CSC population can be enriched after chemotherapy through various mechanisms. In this regard, we recently showed that PI3K/AKT signaling plays a significant role in cisplatin-resistance of CSCs, which is dramatically enriched after treatment [35].

Although TNBCs have a high complete response rate to neoadjuvant therapy, a substantial proportion of patients eventually develop chemo resistance and relapse after treatment. These characteristics are believed to be due to the existence of CSCs, thus targeting abnormal signal pathways to eliminate CSCs might be a promising strategy to manage TNBC (Figure 2). The TGF-β, Notch, Wnt/β-catenin signaling pathways and tyrosine-kinase receptors [9, 36, 37] that regulate EMT and CSCs function are abnormally regulated in TNBC. Thus, we reviewed these pathways plus their regulations, which not only be targeted in the mesenchymal-like TNBCs but also in other TNBCs and serve as potential therapeutic targets.

### Notch signaling pathway

Notch signaling actively regulates normal mammary stem cell self-renewal and differentiation, which is essential for ensuring normal mammary gland development [38, 39]. It is also widely recognized that abnormal expression of Notch pathway members is also involved in breast tumor development. Gallahan et al. showed that the mutagenic insertion of mouse mammary tumor virus (MMTV) generates a truncated and constitutively active form of the Notch1/4 receptor, leading to the formation of mammary tumors in mice [40]. Dysregulation of Notch signaling pathway leads to abnormal self-renewal and transformation of stem cells/progenitors, which undergo aberrant differentiation processes and result in carcinogenesis [41, 42]. Rustighi and coworkers found that activities of Notch1/4 are essential for maintaining the ‘stemness’ character of both normal stem cells and breast cancer stem cells (BCSCs), and that Notch1/4 activity is strongly correlated with self-renewal and chemo resistance of BCSCs [43]. Harrison and coworkers isolated BCSCs from breast cancer cell lines and primary breast cancer samples by sorting cells resistant to anoikis or cells containing markers of ESA^+^/CD44^+^/CD24^low^, and they found that Notch4 is specifically overexpressed in these BCSCs [44]. Furthermore, pharmacological or genetic inhibition of Notch4 signaling markedly reduces BCSC proliferation and self-renewal *in vitro* and tumor formation *in vivo*. In 2013, Reipas demonstrated that p90 ribosomal S6 kinase (RSK) is an activator of Notch4 signal in TNBC by phosphorylation of Y-box binding protein-1 (YB-1), which is an oncogenic transcription factor binding to the Notch4 promoter [45]. Thus *in vitro* kinase assays and molecular docking were employed to screen for RSK inhibitors from off-patent drugs, and a lead candidate luteolin was found. As anticipated, luteolin showed a high ability to block the Notch4 signaling and suppress proliferation of TNBC, especially BCSC-enriched populations. Similarly, Notch1 also plays a key role in the regulation of BCSC. *In vivo* studies showed that the Notch1 signal is responsible for the chemo resistance in TNBC cells after treatment of docetaxel. Furthermore, docetaxel-treated cells are enriched for CSCs and became more tumorigenic when replanted into xenograft models [46, 47]. In contrast, γ secretase inhibitor could reverse the chemo resistance of these cells and diminish the CSCs pool in combination with docetaxel. In a TNBC patient-derived xenograft model, Notch1 monoclonal antibodies exhibited synthetically antitumor efficacy combined with docetaxel *via* inhibition of CSCs [48].

### Wnt/β-catenin signaling pathway

The Wnt receptor frizzled-7 and LRP5/6 have been found to be up-regulated in many TNBCs and are associated with poor prognosis [49, 50]. Lack of β-catenin around the membrane or abundant accumulation of β-catenin in the nucleus is regarded as a surrogate marker of Wnt signaling pathway activation, which is found to be enriched in TNBC and basal-like breast cancer [51]. Compelling evidence indicates that Wnt10B may be a valuable therapeutic target for TNBC, and a transgenic murine model with a Wnt10B-driven tumor is shown to have characteristics of human TNBC [52].

The Wnt/β-catenin signaling is believed to be implicated in the control of various stem cells even from distinct tissues and may act as a niche factor to maintain the self-renewing of stem cells [53–55]. There are reports demonstrating that IHC staining of nuclear β-catenin was overlapped with CD44^+^/CD24^low^ staining [56]. Recently, through high throughput screening, Gupta and colleagues identified salinomycin as a selective inhibitor of CSCs through inhibition of Wnt/β-catenin signaling pathway and degradation of LRP6 [57]. The reduction of β-catenin significantly inhibits tumorigenic ability of TNBC cell lines both *in vitro* and *in vivo*, and the stemness of cancer cells is also reduced [58]. In addition, Johnson has developed a mouse model of breast cancer that mimics TNBC by eliminating of Retinoblastoma (Rb), p53, and BRCA1. These tumors have demonstrated a stem phenotype that can be suppressed by Wnt pathway inhibitors [59].

### Hedgehog signaling pathway

Hh signaling plays important roles in embryonic mammary gland induction and ductal morphogenesis, which is demonstrated by the synergetic regulation of Ptch1 and Gli-2 in mediating epithelial-stromal interactions [60, 61]. Disruption of either Ptch1 or Gli-2 leads to defects in ductal morphogenesis, which suggests a role for abnormal Hh signaling in breast cancer formation. Meanwhile, it has been reported that Hh signaling components were over-expressed in both BCSCs and human normal mammary stem/progenitor cells [62].

In fact, many genes of Hh pathways are known oncogenes, including Gli1/2, Sonic Hedge-hog (Shh) and Smo, and Ptch1 can also be classified as a tumor suppressor. The Ptch1 locus is one of the most commonly detected changes among the tumor suppressor genes, which occur in about 19% of human breast cancers and in up to 33% of breast cancer cell lines [63]. Interestingly, the Gli1 expression is inversely associated with ER expression and Smo/Gli1 expression is significantly higher in the TNBC than non-TNBC forms [64]. Mukherjee reported that Ptch1 and Smo are expressed at low level in the normal tissue, and Smo is over-expressed in nearly 70% of ductal carcinomas and in about 30% of metastasis breast cancer tissues [65].

The role of Hh signaling pathway in the self-renewal of mammary stem cells is well established. Nevertheless, the regulation role of this pathway in BCSCs has yet to be determined [42]. Liu has shown that Hh pathway activation alters the expression of Bmi-1, and further alters tumorigenic potential of BCSCs [62]. Hh pathway members are highly expressed in CD44^+^CD24^−/low^ populations, and inhibition of Hh signaling by cyclopamine or Gli1 siRNA suppresses the proliferation and maintenance of these cells [66].

### TGF-β signaling pathway

Tumor invasion is often associated with EMT, as disseminated cancer cells seem to require the capacities of the stem cells to form secondary tumors. Thus, the EMT process might also confer the self-renewal capability to disseminated cancer cells. In 2008, Sendurai and colleagues reported a direct association between EMT and stem cell properties [67]. They showed that exposure to TGF-β1 dramatically enhanced mammary stem cells proportion and increased their ability to form mammospheres, and the gene expression profile of EMT resembled that of breast cancer stem cells (e.g. N-cadherin, Slug, E-cadherin, and Snail). Furthermore, more differentiated neoplastic cells that undergo EMT process could generate the cancer stem-like cells, as demonstrated by their increased ability to form mammospheres and tumors in mouse hosts [67]. In 2011, Michaelhas reported the generation of CSCs by inducing EMT, which is induced *via* exposure of tumor cells to cytokines (TGF-β/TNFα) [68]. These induced CSC-like cells were equipped with increased self-renewing capacity, more aggressive tumorigenicity and increased resistance to chemo agents. More recently, Deng et al. reported that CD24 expression is regulated by TGF-βR1 signaling, which contributed to chemo resistance in TNBCs [69].

The vital role of TGF-β signaling in EMT induction and subsequent acquisition of stemness has suggested this pathway as a new therapeutic approach to target BCSCs. Indeed, inhibition of TGF-βRI combined with paclitaxel significantly reduced CSC frequency and disease progression in an *in vivo* model [70]. Li has shown that caffeic acid attenuated the CSCs-like properties of CSCs derived from TNBC cell lines by inhibition of TGF-β/Smad2 signaling pathway both *in vitro* and *in vivo* [71].

## IMMUNE ASSOCIATED TNBC

The immunomodulatory TNBC identified by Brian and basal-like immune activated TNBC identified by Matthew are enriched in gene ontologies of immune cell signaling, cytokine signaling, antigen processing-presentation, and core immune signal transduction pathways [9, 10]. It is not surprising that the immune associated TNBC subtype is associated with enhanced levels of immune cell infiltration and resulted in a good clinical outcome, and tumor-infiltrating lymphocytes seem predictive of neoadjuvant chemotherapy response [72–75]. The immune checkpoint is an elaborate machine that prevents the excessive activation of T-cells under normal conditions, and immune checkpoint blockade-based cancer therapies are currently being increasingly evaluated in the clinical studies. Cytotoxic-T-lymphocyte-antigen-4 (CTLA-4) is a cell surface receptor of lymphocyte T regulators and suppresses T-cell activation. Blockage of CTLA-4 via anti-CTLA-4- mAbs (e.g. ipilimumab and tremelimumab) is predicted to enhance T-cell activity against tumor cells and is under clinical trial for breast cancers (NCT02892734 and NCT02563925) [76]. The PD-L1/PD-1 pathway is a potent mechanism by which tumor cells evade host immune surveillance, and anti-PD-1 and anti-PD-L1-mAbs could also enhance T-cell immune response towards tumor cells (clinical trials in breast cancer include NCT02838823 and NCT02129556) [77, 78]. Recently, there has been great interest in investigating immune checkpoint inhibition in combination with novel agents. In 2017, ASCO demonstrated the potency of combined HDAC and PD-1 inhibition in TNBC. However, there is no currently active trial to assess their efficacy in the various stratifications of TNBC.

## LUMINAL ANDROGEN RECEPTOR (LAR) SUBTYPE

The LAR subtype identified by Matthew and Brian is characterized by overexpression of AR and hyper-activation of this pathway [9, 10]. Compared to the rest of the TNBC subtypes, the LAR subtype seems especially resistant to various chemotherapies both *in vitro* and *in vivo*, as judged by retrospective analyses of data derived from clinical trials [79, 80]. While the LAR subtype of TNBC showed high response rate to anti-androgens in preclinical studies and clinical trials.

### Inhibition of androgen receptor

AR belongs to a kind of transcription factors that are activated by the binding of either androgenic hormones, dihydrotestosterone, or testosterone in the cytoplasm and then translocate into the nucleus [81]. The AR and AR targeted genes were over-expressed in one-third of TNBCs detected *via* immunohistochemistry [82]. In the *in vitro* cell culture model, the LAR subtype was shown to depend on AR signaling, as elimination of AR greatly decreased cell viability and tumor growth [9]. Furthermore, the anti-androgen bicalutamide and orteronel are currently under phase II study in AR^+^, ER^−^/PR^−^ metastatic breast cancer patients [83, 84]. Together, these studies provide a strong rationale for further investigation of this therapeutic strategy in TNBC. Meanwhile, some LAR TNBC cell lines were found to harbor an activating mutation in Phosphatidylinositol-4, 5-Bisphosphate 3-Kinase, Catalytic Subunit Alpha (PIK3CA), which illuminates the mechanism of resistance to bicalutamide via a compensatory activation of PI3K signaling. In addition, synergistic activity between bicalutamide and PI3K inhibitors was observed in preclinical studies, and providing a rationale for further clinical trials [9]. More recently, Xiaoxiang et al. (2016) reported that PARP1 and AR expression were positively correlated with each other, and BRCA1 expression is negatively correlated with these. They also demonstrated that inhibition of PARP1 and AR had a strong synergy effect in AR positive TNBCs [85].

Recently, there has been an increasing interest in the study of AR negative TNBC, which mainly falls into the basal-like molecular subtype. This so called quadruple negative breast cancer (QNBC) [86–88] lacks the defined targetable pathways mentioned above, and continuous effort has been made to look for targets that are dysregulated in QNBC. In 2017, the ASCO annual meeting showed an investigation of AR expression in TNBC patients. They found that AR expression was positive in 30% of TNBC, and AR negative is significantly associated with the younger age group, higher grade, and higher tumor stage.

### Phosphatidylinositol-4, 5-bisphosphate 3-kinase, catalytic subunit alpha

In the last decades, various high throughput analyses in TNBC have discovered a spectrum of gene alteration. Among them, TP53 is the most frequently mutated locus (62%), followed by PIK3CA mutation (10%) [89]. At the same time, the PIK3CA activating mutations seem to be enriched in the LAR molecular subtype of TNBC [9], which sensitize this subtype of TNBC to combined AR and PI3K inhibition [90]. When targeted clinically, patients with PIK3CA mutations showed a favorable response to PI3K signaling inhibition [91]. Besides activating mutations of PIK3CA, alterations in INPP4B (function as an antagonist of PI3K/AKT signaling) and PTEN were also found to frequently occur in TNBC [92], making the PI3K/AKT/mTOR pathway an attractive intervention point for a large fraction of TNBC cases.

## OVERLAPPING THERAPEUTIC TARGETS

Tremendous efforts have been made in the identification and characterization of therapeutic targets for TNBC in the past years. Some of these targets have been entered in difference phases of clinic trials (Table 1). If successful, these studies should greatly benefit the long-term survival of cancer patients.

**Table 1:** Widely studied therapeutic targets that under investigations in the clinical trial for TNBC

Therapeutic targets	Drug	Mechanism of action	Phase	Patient population	Regimen	ClinicalTrials. gov ID	Reference
EGFR	Afatinib	Pan-ErbB dimers inhibitor	Phase II	TNBC	+Paclitaxel	NCT02511847	
	Gefitinib	EGFR TKI	Phase II	TNBC with EGFR positive	Monotherapy	NCT01732276	[97, 98]
	Cetuximab	EGFR-mAb	Phase II	Breast Cancer contains TNBC	+Ixabepilone	NCT01097642	[99, 100]
	MM 151	Oligoclonal anti- EGFR antibody	Phase I	Advanced solid tumor contains TNBC	+Irinotecan	NCT01520389	
	Lapatinib	EGFR/HER2 TKI	Pilot Study	Metastatic TNBC	+Veliparib	NCT02158507	
VEGF/VEGFR	Bevacizumab	VEGF-A inhibitor	Phase II	TNBC	+Doxorubicin/ Temsirolimus	NCT02456857	[102–107]
	Cediranib	VEGFR inhibitor	Phase II	Solid tumors contain TNBC	+Olaparib	NCT02498613	[26]
AR	GTx-024	Selective androgen receptor modulator	Phase II	TNBC with AR positive	Monotherapy	NCT02368691	
	Orteronel	antiandrogen	Phase II	Metastatic breast cancer	Monotherapy	NCT01990209	[84]
	Bicalutamide	AR inhibitor	Phase II	TNBC with AR positive	+Physician's Choice	NCT02353988	[9, 83]
PI3K/AKT/ mTOR	GSK2141795	AKT kinase inhibitor	Phase I	Cancer contains TNBC	+MEK inhibitor	NCT01138085	
	BKM120	PI3K inhibitor	Phase II	TNBC	Monotherapy	NCT02000882	
	AZD5363	AKT kinase inhibitor	Phase I	Cancer contains TNBC	+olaparib	NCT02338622	
PARP	Iniparib	PARP inhibitor	Phase II	TNBC	+paclitaxel	NCT01204125	[18, 27]
	Olaparib	PARP inhibitor	Phase I	Cancer contains TNBC	+AZD5363	NCT02338622	[25, 26, 28]
			Phase II	Solid tumors contain TNBC	+Cediranib Maleate	NCT02498613	
	Talazoparib	PARP inhibitor	Phase III	Breast cancer patients with BRCA mutation	+ Physician's- Choice	NCT01945775	
FGFR	Lucitanib	FGFR and VEGFR inhibitor	Phase II	Metastatic breast cancer	Monotherapy	NCT02202746	
Notch pathway	PF-03084014	Gamma-Secretase inhibitor	Phase II	TNBC	Monotherapy	NCT02299635	[46, 47]
	RO4929097	Gamma-Secretase inhibitor	Phase I	Breast cancer contains TNBC	+Vismodegib	NCT01071564	
Wnt/β-catenin pathway	Foxy-5	Small peptide mimicking Wnt-5a	Phase I	Metastatic breast cancer	Monotherapy	NCT02020291	[50]
Hedge-hog pathway	LDE225	Smo antagonist	Phase I	TNBC	+Docetaxel	NCT02027376	
TGF β pathway	Fresolimumab	TGF β-mAb		Metastatic breast cancer	+Radiation therapy	NCT01401062	
	LY2157299	TGFβR1 kinase inhibitor	Phase II	Metastatic breast cancer	+Radiation therapy	NCT02538471	
CTLA-4	Ipilimumab	anti-CTLA-4- mAbs	Phase II	HER2 negative Breast Cancer	+Nivolumab	NCT02892734	
	Tremelimumab	anti-CTLA-4- mAbs	Recruiting participants	Metastatic Breast Cancer	+HER2 directed therapy	NCT02563925	[76]
PD-1	JS001	anti-PD-1- mAbs	Phase I	TNBC	Monotherapy	NCT02838823	
	Pembrolizumab	anti-PD-1- mAbs	Phase I	TNBC	+Radiation	NCT02977468	[77, 78]

Drugs for each target are listed with its mechanism of action, and patients’ condition and treatment regimen are listed below.

### Epidermal growth factor receptor (EGFR)

EGFR was first identified as an important therapeutic target in lung cancer, and it is also a negative prognostic factor for TNBC [93]. It was reported that approximately half of TNBC cases exhibit EGFR expression, and EGFR signaling amplification is common in this aggressive form of breast cancer [94, 95]. Teng et. al. investigated a cohort of 653 TNBC patients and showed that up to 11% of the patients had activating mutation in EGFR locus and were sensitive to EGFR-tyrosine kinase inhibitor (TKI) [96]. However, many studies of clinical trials so far have been disappointing. Gefitinib, a small-molecule against EGFR, was shown to have minimal activity in a phase II trial for treatment of metastatic breast cancer [97] and have modest activity in combination with standard chemotherapy [98]. Some clinical trials investigated EGFR monoclonal antibody in combination with platinum agents for the treatment of TNBC, but failed to achieve improved outcomes [99, 100]. In a phase II trial of cetuximab in combination with carboplatin among TNBC patients, this combination produced responses only in fewer than 20% of patients [99]. In these trials, although the majority of patients harbor EGFR pathway activation, cetuximab failed to block the expression of EGFR pathway in most cases, suggesting that alternative mechanisms were involved. In 2013 Yi et al. reported that the effect of EGFR kinase inhibitors in TNBC is attenuated by activation of PI3K/AKT pathway, and combined inhibition of EGFR and PI3K is warranted [101]. More importantly, in some randomized clinical trials, lack of biomarkers to stratify the heterogeneous tumor accurately and identify sensitive patients who are most likely to respond to EGFR blocking remains a major reason for these disappointing results.

### Vascular endothelial growth factor receptor and angiogenesis (VEGFR)

Like EGFR, the VEGFR has also been studied as a therapeutic target for patients with TNBC. Bevacizumab, a humanized monoclonal antibody of VEGFA, has been evaluated in several phase III trials for the treatment of metastatic breast cancer. Two of these phase III trials investigated the curative effect of bevacizumab in a pooled subset of 621 patients [102, 103]. Although bevacizumab has showed improvements in progression-free survival (PFS) of patients, there is no overall survival benefit in the TNBC subgroup [104, 105]. Several phase III trials demonstrated that bevacizumab was ineffective in unselected TNBC patients, including the latest results of phase III Beatrice trial (failure in PFS) [106, 107]. Meanwhile, another newer antiangiogenic agent ramucirumab (IMC-1121B, ImClone) was under investigation. In combined therapy, ramucirumab and docetaxel is currently under phase III trial designed for breast cancer patients with negative HER2 expression (Clinical trials ID: NCT00703326).

Similarly, VEGFR TKIs were also widely assessed in patients with intractable breast cancer. There is little preclinical data that indicates the value of targeting the VEGF pathway in TNBC. The initial evidence came from a phase II trial of a VEGFR TKI (sunitinib), and the overall response to monotherapy was significantly higher in TNBC than in heavily pretreated patients [108]. These results led to the recruitment of a phase III trial via a combination of sunitinib to capcecitabine, but no improvement in PFS of TNBC patients was observed [109].

### Fibroblast growth factor receptors (FGFRs)

The aberrant signaling transduced by fibroblast growth factor receptors (FGFRs) is believed to be involved in the pathogenesis of multiple cancer types. However, compared to other receptor tyrosine kinases, targeting the FGFR signaling as a therapeutic strategy for cancer patients has lagged. FGFR1 and FGFR2 were amplified in about 9% and 4% of TNBC, respectively [2, 110]. Genome-wide association studies also identify FGFR2 as one of the major novel susceptibility loci for breast cancer formation [111, 112]. Preclinical study demonstrated that breast cancer cell lines with FGFR amplification were sensitive to FGFR inhibitor PD173074 [110]. Moreover, dovitinib, a pan-FGFR TKI, was found to have an anticancer effect on breast cancer in preclinical studies [113]. The novel anticancer drug NVP-BGJ398 is a selective FGFR inhibitor, and oncogenic FGFR1 amplification serves as a biomarker for cancer cells and predicts sensitivity to NVP-BGJ398 administration [114].

## OTHER THERAPEUTIC TARGETS FOR TNBC

Beyond these intensively studied targets above, vast efforts have been made to find new therapeutic targets (Table 2) [115, 116]. Wael et al. showed geminin, which is over-expressed in TNBC, is activated by tyrosine kinase c-Abl [117]. Inhibition of c-Abl by imatinib/nilotonib caused tumor recession. Chinois's study (2009) demonstrated that the HSP90 inhibitor PU-H71 is a potential chemotherapy in a xenograft model, and induced complete responses in triple-negative breast cancer [118, 119]. To identify new therapeutic targets in TNBC, Toker performed a short hairpin RNA screening for protein kinases (about 26 kinases) commonly dysregulated in breast cancer [120]. They identified AKT3 as a gene preferentially required for the growth of TNBCs, and inhibition of AKT3 significantly abolished cell growth in three-dimensional spheroid culture model. Semenza et. al. reported that hypoxia-inducible factors (HIFs) signaling is essential for maintenance of BCSCs, and HIFs inhibitors combine with cytotoxic chemotherapy showed favorable synergy [121].

**Table 2:** Some other therapeutic targets under investigations in the clinical trial for TNBC

Therapeutic targets	Drug	Mechanism of action	Phase	Patient population	Regimen	ClinicalTrials.gov ID	Reference
Tyrosine kinase	SU011248	Inhibitor of PDGFR/VEGFR/KIT/FLT3	Phase II	TNBC	Monotherapy	NCT00246571	
MET	Tivantinib	MET inhibitor	Phase II	Metastatic breast cancer	Monotherapy	NCT01575522	
	XL184	MET and VEGFR2 inhibitor	Phase II	TNBC	Monotherapy	NCT01738438	
Src tyrosine kinase	Dasatinib	Src/c-kit/PDGFR inhibitor	Phase II	TNBC	Monotherapy	NCT00817531	[115, 116]
HSP90	AT13387	HSP90 inhibitor	Phase I	TNBC	+paclitaxel	NCT02474173	
	Ganetespib	HSP90 inhibitor	Phase II	Breast cancer contains TNBC	Monotherapy	NCT01677455	[119]
STAT3 pathway	Ruxolitinib	JAK1/2 inhibitor	Phase I	Recurrent breast cancer	Monotherapy	NCT02041429	
TP53/WEE1 pathway	MK-1775	WEE1 inhibitor	Phase I	Advanced solid tumors	Carboplatin/Paclitaxel	NCT02341456	
Nuclear export comptlex	Selinexor	CRM1 inhibitors	Phase II	TNBC	Monotherapy	NCT02402764	

Drugs for each target are listed with its mechanism of action, and patients’ condition and treatment regimen are listed below.

## CONCLUSION AND FUTURE ASPECTS

We have discussed the potential therapeutic targets and related signaling pathways for TNBC based on its intrinsic subtypes. Among these known subtypes, the Basal-like TNBC (especially those harboring mutations or dysfunctions in BRCA1) is quite outstanding, which is believed to be sensitive to platinum compounds and PARP inhibitors. Besides the Basal-like subtype, the LAR TNBC is also quite notable because it is reliably identified by both Brian and Matthew. Despite lacking ERs and PRs, the LAR subtype is enriched in hormonally regulated pathways, which imply a targeted therapy for AR signaling. Indeed, bicalutamide showed clinical benefit in ER/PR-negative and AR-positive breast cancers [83], and enzalutamide is under trial for metastatic AR-positive breast cancers [122].

Managing intractable breast cancer via the development of precision medicine has drawn quite a degree of scientific interest, and this concept also holds great promise to TNBC [123, 124]. The alleged scientific and logistical challenges are the main obstacle that hinders clinical implementation of precision medicine. Future studies should be directed towards identifying novel driver mutations by bioinformatics combined with assessment of pathway activation, and to employ more precise medicines for targeting key driver mutations using more reliable and efficient approaches [125]. In this regard, a recent study by Bruna et al. (2016) developed and studied breast-cancer-patient-derived xenografts (PDTXs) and PDTX-derived cells cultures (PDTCs). The comparison between PDTXs and PDTCs revealed that both models share a similar feature in that they both preserve the intra-tumor heterogeneity of the original tumors and can be used for drug screening. Their study further demonstrated that drugs identified using PDTCs were also highly effective for the treatment of PDTXs. This leads to a promising application that PDTCs may serve as a robust platform for pre-clinical pharmacogenomics studies [126].

Future studies should also be directed at identifying more subtypes of TNBC based on their specific features of DNA, RNA, protein, and epigenetics. Given the intrinsic molecular and clinical heterogeneity of TNBC, it is highly possible to further classify it into more different subtypes. Targeted therapies developed against these specific subtypes should greatly benefit patient care and treatment, and effectively extend the health life of these patients.
